# Impacts of Low Temperature on the Teleost Immune System

**DOI:** 10.3390/biology6040039

**Published:** 2017-11-22

**Authors:** Quinn H. Abram, Brian Dixon, Barbara A. Katzenback

**Affiliations:** Department of Biology, University of Waterloo, 200 University Ave West, Waterloo, ON N2L 3G1, Canada; qhabram@uwaterloo.ca (Q.H.A.); bdixon@uwaterloo.ca (B.D.)

**Keywords:** teleosts, temperature, innate immunity, adaptive immunity, cytokines, macrophages, major histocompatibility class I, antigen presentation, lymphocyte proliferation, antibodies

## Abstract

As poikilothermic vertebrates, fish can experience changes in water temperature, and hence body temperature, as a result of seasonal changes, migration, or efflux of large quantities of effluent into a body of water. Temperature shifts outside of the optimal temperature range for an individual fish species can have negative impacts on the physiology of the animal, including the immune system. As a result, acute or chronic exposure to suboptimal temperatures can impair an organisms’ ability to defend against pathogens and thus compromise the overall health of the animal. This review focuses on the advances made towards understanding the impacts of suboptimal temperature on the soluble and cellular mediators of the innate and adaptive immune systems of fishes. Although cold stress can result in varying effects in different fish species, acute and chronic suboptimal temperature exposure generally yield suppressive effects, particularly on adaptive immunity. Knowledge of the effects of environmental temperature on fish species is critical for both the optimal management of wild species and the best management practices for aquaculture species.

## 1. Introduction

The impact of temperature changes on biological systems is an important topic in relation to global climate change and differences in seasonal temperatures. Higher frequencies and magnitudes of extreme temperature events such as cold snaps are occurring due to increasing climate variability [1,2]. The unprecedented cold-weather experienced in the Gulf of Mexico in 2010 is one example of these extreme temperature events and resulted in a 12°C and 6°C drop in air and water temperatures, respectively, over a two week period leading to widespread mortality in fish populations [3]. Furthermore, variance in seasonal water temperatures that fishes experience within a given year can be quite large, ranging from below 5°C to 19°C for a cold water species such as rainbow trout, and 16°C to 39°C for zebrafish, a warm water species [4,5,6]. The poikilothermic nature of fish requires special consideration in the face of these challenges as changes in water temperature equate to changes in body temperature and can impact key physiological processes, such as the immune system and ultimately the health of the animal [7,8].

While temperatures above the physiological range of a fish species triggers a stress response that can negatively impact immune function [9,10], so too can suboptimal temperatures have a negative impact on fish immunity and health. For example, water temperature is one factor when considering whether to transfer Atlantic salmon smolts to ocean pens as prevalence of the parasite *Kudoa thyrsites* on these fish was greatest when water temperatures were above 10°C in the summer and fall, and not detected in the winter and spring when the water temperature was below 10°C [11]. In contrast, a number of fish-pathogen model systems exist in which an immunosuppressive effect of suboptimal temperatures is observed. For instance, olive flounder (*Paralichthys olivaceus*) is susceptible to viral haemorrhagic septicaemia virus (VHSV) at hypothermic temperatures such as 15°C with 24% mortality, whereas mortality is not observed when fish are maintained at 20°C [12]. Tilapia (*Oreochromis niloticus*) infected with *Streptococcus iniae* [13] or orange-spotted grouper (*Epinephelus coioides*) infected with *Vibrio alginolyticus* [14] suffered significant mortalities when placed in water that was 8°C below (or above) their thermal optimum. In cyprinids, infection of zebrafish with spring viraemia carp virus (SVCV) results in higher mortality rates when fish are kept at suboptimal temperatures [15]. As a final example, a natural model system exists which exemplifies the effects of temperature on the immune system and disease outcome in fish—the walleye, *Sander vitreus*, and walleye dermal sarcoma virus (WDSV) that causes cutaneous mesenchymal neoplasms [16]. Tumor progression has a seasonal cycle: appearing in late fall, persisting into early spring and then regressing in the summer [17], suggesting a link with temperature [16]. Once walleye recover in the spring/summer, they appear to develop immunity to WDSV [16]. It is hypothesized that cold stress negatively impacts walleye immunity and allows for virus proliferation, tumor formation and virus transmission. The aforementioned interactions between fish, pathogens and temperature speak to the complex interplay occurring that may result in compromised fish health. It is important to note that increases in pathogenesis at low temperatures may also be partially due to the effect of temperature on the virulence of the pathogen, which was reviewed by Guijarro et al. [18]. Herein we review the impact of hypothermic conditions on the innate and adaptive immune system of teleosts. 

## 2. Innate Immunity and Temperature

### 2.1. Components of Innate Immunity

Like that of mammals, the teleost immune system consists of innate and adaptive arms (discussed below) [19,20]. The innate arm is constitutive and consists of germ-line encoded effector molecules (antimicrobial peptides, complement proteins) and cells (macrophages, neutrophils, basophils, eosinophils, cytotoxic cells) that recognize conserved microbial associated molecular patterns (MAMPs) [19,20,21]. Innate immune cells, such as macrophages and neutrophils, also produce hundreds of bioactive molecules that direct the initiation and resolution of an inflammatory response and are critical to the survival of an organism [22,23]. In particular, macrophages are arguably the central innate immune cell and recognize, uptake (phagocytosis) and neutralize pathogens and act to bridge the innate and adaptive arms by driving T cell responses through antigen presentation.

### 2.2. Complement

Complement proteins are the major soluble component of the innate immune system and consists of approximately 30 proteins that collectively comprise the classical, lectin and alternative complement pathways, reviewed in [24]. All three pathways lead to the formation of the membrane attack complex (MAC) that is cytolytic to target cells/microbes as well as the release of complement cleavage products that play a role in inflammation [24]. Despite being a prominent and essential component of immunity, few studies have examined the effect of suboptimal temperature on complement levels and activity. One study examined the short term exposure of tilapia (*Tilapia zillii*) to cold stress (17°C for 30 min) and found there was no difference in serum complement activity from these fish compared to control fish housed at 27°C [25]. However, it is difficult to ascertain the relevance of this particular study due to the short duration of thermal stress and the single fish species that was examined. Although few studies have investigated chronic exposure of fishes to cold stress, the studies conducted reveal conflicting responses of complement activity from different fish species. For example, lowered opsonization capacity and lytic activity of serum complement from rainbow trout acclimated to lower temperatures (5°C) over a period of greater than two months was observed compared to those fish maintained at higher temperatures (>10°C) [26]. In contrast, sockeye salmon possessed higher serum complement activity when reared at 8°C versus 12°C [27]. The underlying mechanisms of complement regulation in these two fish species, both from the family *Salmonidae*, are unknown and suggest that even within a family of fish, suboptimal temperatures have differing effects on complement activity.

Studies investigating the impact of cold stress on complement molecule regulation in immunostimulated or infected fish are largely lacking. A study on rainbow trout demonstrated upregulation of C5a receptor transcript levels in the spleen and kidney of *Yersinia ruckeri* immunized (i.p.) animals regardless of temperature (5°C, 15°C or 25°C) [28], suggesting that cold stress may not impact upregulation of the complement system during pathogen challenge. However, further studies are needed in order to assess the impact of temperature on the complement system.

### 2.3. Leukocyte Numbers

The availability and proportion of leukocytes in the blood, kidney and spleen (the major immune organs of teleosts) are important indicators of the immunocompetence of an animal. In some cases, hypothermic temperatures had no effect on packed cell volume or percentage of blood leukocytes, such as in Atlantic halibut (*Hippoglossus hippoglossus* L.) (8°C versus 12°C–15°C) [29], or in the total number of anterior kidney neutrophils in channel catfish (10°C versus 24°C) [30]. Similarly, the percentage of monocytes, thrombocytes and granulocytes in the peripheral blood leukocyte (PBL) population or spleen did not differ between rainbow trout maintained at 12°C compared to 15°C [31]. However, study of other fish species provides contrasting results. In carp (*Cyprinius carpio*), circulating granulocyte numbers double in the blood, coinciding with a decrease in kidney granulocyte numbers, in response to acute hypothermic stress [32], while sockeye salmon reared at 8°C versus 12°C tended to have a higher percentage of phagocytic kidney macrophages and a decrease in peripheral blood lymphocytes [27]. Yet, members of the order Perciformes exhibit a decrease in the total blood leukocyte numbers as observed in orange-spotted grouper (*Epinephelus coioides*) [14], tilapia (*Oreochromis mossambicus*) [13] and hybrid striped bass (*Monrone chrysops* × *Morone saxatilis*) at suboptimal temperatures [33], with hybrid striped bass also showing a reduction in blood monocyte numbers [33]. These data suggest that hypothermic temperatures impact orders of fishes differentially under non-challenged conditions.

The impact of suboptimal temperature on rainbow trout challenged with bacterial and protozoal pathogens revealed that leukocyte composition was affected by temperature during infection. Upon challenge with *A. salmonicida*, an increase in the percent of granulocytes in the PBLs, but not in the monocyte or thrombocyte population, was observed in rainbow trout maintained at 12°C. However, a much greater increase was observed in granulocyte numbers, as well as monocyte numbers, in the PBLs isolated from *A. salmonicida* challenged fish maintained at 15°C [31]. A similar result was observed in the number of monocytes in the spleen in which greater numbers were present in fish maintained at 15°C versus 12°C following *A. salmonicida* infection [31]. While the percentage of leukocytes in the peripheral blood of rainbow trout infected with the parasite *Tetracapsuloides bryosalmonae* also increased over time at both 12°C and 15°C, the increase was significantly greater at the higher temperature at all time points [34]. These data suggest that while innate immune cells can be actively mobilized during periods of low temperature, their maximal mobilization is dampened compared to those fish maintained at homothermic temperatures and may predispose these fish to infection.

### 2.4. Peripheral Blood Leukocyte Function

Examination of PBLs from fish exposed to acute or chronic hypothermic temperatures showed a generalized suppression in activity. The phagocytic index of blood leukocytes isolated from orange-spotted grouper (*Epinephelus coioides*) was reduced after 24 h, 48 h and 96 h at 8°C below their thermal optimum [14]. Similar results were observed in a study with tilapia (*Oreochromis mossambicus*) [13]. The phagocytic activity of rainbow trout blood leukocytes was also significantly reduced in rainbow trout acclimated to 5°C for over 2 months (versus 10°C or 15°C) [26]. The production of reactive oxygen species (ROS) was impaired in channel catfish [35], orange-spotted grouper [14], tilapia (*Oreochromis mossambicus*) [13] and rainbow trout [26] PBLs under hypothermic conditions. Despite pathogen challenge with *A. salmonicida*, rainbow trout maintained at a lower temperature (12°C versus 15°C) exhibited suppressed non-specific responses [31]. In particular, splenocytes and PBLs isolated from *A. salmonicida* challenged fish maintained at 15°C cleaved tetrazolum salts to a greater extent than those maintained at 12°C, suggesting an impairment in cell activation or metabolism at lower temperatures [31]. Thus, it appears that acute and chronic cold stress compromises phagocytic activity and ROS production, suggesting a potential impairment of critical innate immune cell function for pathogen destruction.

### 2.5. Cytotoxic Cells

Fish cytotoxic cells are the precursors or equivalent of natural killer (NK)-cells in mammals, appearing to be morphologically distinct, but functionally similar as they have been shown to induce cytotoxicity in mammalian tumor cells and in fish protozoal parasites [19]. Studies on carp cytotoxic cells suggest there is an enhancement of killing activity of cytotoxic cells from fish maintained at hypothermic temperatures. In the first study, carp (*Cyprinus carpio*) were maintained at 25°C, but also acclimated to 10°C for varying durations up to 112 days. Natural killer-like cells were isolated from the kidney and used in a cytotoxicity assay that employed human K562 cells as target cells [36]. NK-like cells from carp maintained at 25°C had higher cytotoxic activity when the *in vitro* NK cell killing assay was performed at 25°C versus 10°C. Similarly, NK-like cells isolated from fish that had been maintained at 10°C for extended periods exhibited higher cytotoxic activity in *in vitro* NK cell killing assays when they were performed at 10°C compared to 25°C [36]. This study suggests that fish innate immune cells are capable of adjusting to long-term hypothermic conditions in which these new temperature conditions are adapted to as normothermic conditions thus allowing NK-like cells to maintain their function [36]. In addition, this study illustrated the importance of selecting appropriate *in vitro* assay conditions (i.e., temperature) for assessment of *ex vivo* cell functions. Similar results were obtained by another group that observed an enhancement of cytotoxic cell activity from carp maintained at 12°C (versus 20°C) for 28 or 42 days [37,38]. However, cytotoxic activity returned to baseline levels by 56 days post transfer of fish to 12°C from 20°C [37]. Interestingly, and in contrast with the aforementioned study, at no time point examined was there a suppression in cytotoxic cell activity compared to those cytotoxic cells from carp maintained at 20°C [37]. Furthermore, activity of cytotoxic cells from cold acclimated fish could not be enhanced *in vitro* if placed at 12°C [38]. These studies suggest that cytotoxic cells are able to adapt to cold temperatures *in vivo*, at least in cyprinids, and may compensate for other aspects of fish immunity that may be negatively impacted by hypothermic temperatures.

### 2.6. Macrophages and Granulocytes

Macrophages and neutrophils are central innate immune cells and are important phagocytic cells as well as producers of reactive oxygen species, amongst an array of antimicrobial arsenal [39,40,41]. Studies on the impact of suboptimal temperatures on macrophages and neutrophils (granulocytes) have generally revealed either no change in their activity or an enhancement of activity. There was no significant difference in the respiratory burst activity of rainbow trout macrophage isolated from the kidney in response to macrophage activating factor (MAF) under low temperatures conditions *in vitro* [42]. Meanwhile, rainbow trout neutrophils had reduced ROS production in response to phagocytic stimuli at lower *in vitro* temperatures [43]. However, the aforementioned studies were performed *in vitro* and thus may not be reflective of *in vivo* conditions. *In vivo* studies on pronephros macrophages isolated from carp (*Cyprinus carpio*) maintained at 12°C (versus 20°C) for 28 days had both a higher respiratory burst response and phagocytic index [44]. Similarly, tench (*Tinca tinca* L.) blood granulocytes from fish maintained at 12°C (winter temperatures) displayed greater phagocytic capacity and production of superoxide anions than that of blood granulocytes from fish maintained at 22°C [45]. These studies suggest that the cellular components of the innate immune system may be enhanced during long-term exposure to hypothermic temperatures thus allowing for compensation in other areas of immune deficits.

### 2.7. Expression of Genes Involved in a Proinflammatory Response

The effects of hypothermic temperatures on the expressions of various immune genes including proinflammatory cytokines, antiviral pathway proteins and Toll-like receptors (TLRs) during fish ontogeny have been most studied in zebrafish. During zebrafish ontogeny, studies suggest a suppression of *il1b*, *tnfa*, *ifn1*, *ifng*, *inos*, *irf3*, *mda5* and *mx* expressions at suboptimal temperatures (15°C compared to 28°C), while the expression pattern of *tlr3* [46], *tlr21* and *tlr22* [47] remained unchanged. Additionally, in Atlantic salmon parr, gene expression of *mx* was delayed at 6°C relative to 14°C following poly I:C injection, but was longer lasting, suggesting that lower temperatures decrease the kinetics of this particular response and perhaps others, but do not eliminate the response completely [48]. Furthermore, developing zebrafish revealed their use as a temperature-dependent model of anemia. Zebrafish raised at cooler temperatures (17°C versus 26.5°C) for up to 7 months display a selective decrease in erythrocytes, but not myeloid cells, resulting in an anemic state [49]. Examination of the gene expression of key growth factors involved in hematopoiesis revealed a decrease in the expression of erythropoietin (*epo*) and erythropoietin receptor (*epor)* in kidney marrow, important for erythropoiesis [50,51,52], but not in colony-stimulating factor-1a (*csf1a)* or *csf3* expressions that are important for generation of macrophages and granulocytes, respectively [49,53,54,55]. These studies suggest that while macrophages and granulocytes (neutrophils) are still produced at normal levels, the expressions of key proinflammatory cytokines and proteins involved in viral recognition may be suppressed or delayed, at least at the transcript level, under hypothermic levels during fish ontogeny.

In adult fish, hypothermic temperatures appear to suppress or delay the production of key innate immune molecules in fish in response to pathogen mimics or pathogens themselves, suggesting a block in the induction of a proinflammatory or antiviral response. For example, rainbow trout head kidney leukocytes treated with lipopolysaccharide (LPS) *in vitro* displayed higher *il1b* mRNA levels at 22°C than at 14°C and transcription of *il1b* mRNA was completely blocked at 4°C [56]. *In vivo* experiments tend to follow a similar trend. In sevenband grouper (*Epinephelus septemfasciatus*) injected with poly I:C, inducible expression of *mx* transcripts in the head kidney were lower in fish maintained at 15°C and 20°C compared to those fish maintained at 25°C [57]. In rainbow trout immunized (i.p.) with *Yersinia ruckeri*, *il1b* and *ifng* transcript levels were upregulated in the spleen and kidney regardless of temperature (5°C or 15°C) [28], however, the upregulation of the proinflammatory transcripts occurred slightly faster at the optimal temperature [28]. Furthermore, only *il10* transcripts were induced following *Y. ruckeri* vaccination of rainbow trout maintained at 15°C, but not at 5°C, while no changes were observed in *tgfb* transcript levels in fish maintained at either temperature [28]. However, the route in which rainbow trout were vaccinated with *Y. ruckeri* impacted whether temperature dependent effects on proinflammatory cytokine expression were observed – bath vaccination of rainbow trout did not produce the same results as that of intraperitoneal vaccination [58]. Despite differing results in regulation of proinflammatory transcript levels, fish that were bath vaccinated with *Y. ruckeri* and maintained at 15°C were able to survive subsequent homologous challenge infection whereas fish at 5°C were not [58]. These data suggest that resolution of *Y. ruckeri* infection in trout is temperature dependent and, although the mechanism of protection remains to be fully elucidated, it appears that production of proinflammatory cytokines plays a role [58].

### 2.8. Antigen Presentation Pathway

Based on studies in mammals, antigen processing and loading for major histocompatibility complex (MHC) I and II occurs through a complex process guided by accessory molecules. In the endogenous pathway, cytosolic proteins are digested by the proteasome into peptides [59], which are then transported into the lumen of the endoplasmic reticulum (ER) by the transporter associated with antigen processing (TAP) where they will be loaded into the peptide binding groove of MHC I [60]. The nascent MHC class I heavy chain is synthesized by ribosomes along the rough ER and secreted into the ER. MHC I heavy chain is unstable and is stabilized by binding to the chaperone calreticulin [61]. The small subunit of the receptor, beta-2 microglobulin (β_2_M), joins this complex and the calreticulin is replaced by calnexin [61]. This complex is recruited to TAP by the chaperones ERp57 and tapasin [61]. Tapasin assists in loading the peptides from TAP into the peptide binding groove of MHC I [61]. Once the appropriate peptide is loaded, the MHC I heavy chain: beta-2 microglobulin: peptide trimer is stable and can shed the chaperones and traffic to the cell surface to present the peptides to CD8+ T cells.

In the exogenous pathway, nascent MHC II alpha and beta chains are also synthesized along the rough ER and fold together in the lumen with the assistance of the MHC II Associated Invariant Chain (Ii), which also blocks the peptide binding groove of MHC II to prevent binding of endogenous peptides in the ER lumen [62]. Ii traficks the MHC II into endosomes that then fuse with lysosomes containing peptides produced from exogenous proteins that the APC has internalized by phagocytosis, pinocytosis or receptor mediated internalization. Here the Ii is proteolytically degraded until only the Class II associated Invariant Peptide (CLIP) remains bound in the peptide binding groove and another chaperone, DM, facilitates the replacement of CLIP with the exogenously derived peptides [62]. Once again, when a stable trimer comprising the two MHC polypeptide chains and a peptide is formed, the complex is stable and moves to the cell surface to present antigen to CD4+ T cells. Antigen presentation to T cells initiates and directs the type of resulting adaptive immune response: a cell-mediated response or an antibody-mediated response. Clearly, the initiation of an adaptive immune response by phagocytes for defense against pathogens greatly depends on antigen presentation. 

Studies have shown cold stress to influence antigen presentation in fish. In carp, cell surface MHC I was downregulated at 6°C (versus 12°C), and appeared to be due to decreased β_2_M mRNA transcription [63]. However, in cold-adapted fish species such as rainbow trout and Atlantic salmon, β_2_M is still synthesized by cells and is trafficked to the surface along with MHC I at 2°C, suggesting that these fish species may be adapted for detecting viruses at low temperatures, contrary to mammals and other fish species [64]. Subsequent studies, however, have shown that there is no accumulation of β_2_M in the media of stimulated or nonstimulated cells cultured at 2°C, suggesting that while the MHC I receptor is present on the cell surface, it is not functional [65]. Conversely, MHC II expression is downregulated at 2°C—but not 5°C—in rainbow trout cells, suggesting a susceptibility to bacterial diseases during cold stress [66]. Recent studies have identified, characterized and produced polyclonal antibodies to rainbow trout genes involved in the antigen processing pathway (APP) including MHC I [67], β_2_M [64], TAP1/2 [68] tapasin [69], calreticulin [70,71], ERp57 [72] and calnexin [73]. Collectively, these studies demonstrate the largely conserved nature of the APP in rainbow trout. Studies have also examined the regulation of the APP in response to viral infection with viral hemorrhagic septicemia virus (VHSV) in conjunction with cold [65]. These data demonstrate an increase in the protein levels of MHC I, β_2_M and tapasin in rainbow trout cells infected with VHSV at 14°C [65]. As previously reported, MHC I and β_2_M protein levels do not change with cold stress [65]. If, however, cells were infected at 2°C (cold stress), the VHSV-infected cells failed to upregulate protein levels of MHC I, β_2_M and tapasin [65], suggesting cold stress has a negative impact on antigen presentation, and leads to an impaired immune response when challenged with a pathogen.

Using a newly developed Arctic char cell line, Semple et al. [74] observed that there was also no difference in the protein levels of MHC I and β_2_M at 1, 4 or 14°C in nonstimulated cells. Arctic charr are particularly adapted to very cold temperatures and thus may have evolved mechanisms to maintain antigen presentation pathways in the cold. Interestingly for this cell line, MHC I, β_2_M and ERp57 protein levels did not increase with poly I:C treatment at 14°C, suggesting that the regulation of the Arctic charr antigen presentation pathway differs in response to temperature from that of other salmonids, perhaps due to their adaptation to colder temperatures. The lack of MHC I, β_2_M and ERp57 protein level upregulation was specific to the APP and not an impairment of immunity altogether, as Mx protein was induced by doses of poly I:C as low as 10 µg/mL in 24 h, at 14°C, and in cells exposed to 1°C or 4°C within 7 days at a dose of 50 µg/mL. While there are general trends in the effects of temperature on immunity in teleost fish and specifically within the salmonids, there are species–specific differences that have likely evolved as adaptations to the environment that these species inhabit. Thus, while there have been some preliminary studies, the effects of thermal stress on APP function and MHC trafficking are largely unknown and represents a knowledge gap in terms of how antigen presentation is impacted in fish species facing increased magnitude and fluctuations in environmental temperatures.

## 3. Adaptive Immunity and Temperature

### 3.1. Components of Adaptive Immunity

The adaptive arm of the vertebrate immune system is inducible, pathogen-specific, and generally results in immunological memory by cellular (B cells, T cells) and humoral (antibodies) components [19,20]. T lymphocytes recognize antigens presented by cells expressing MHC I and II and, upon recognition, induce specific cytotoxicity or release cytokines that act on other lymphocytes and innate immune cells to direct a specific response against a pathogen respectively [75]. B lymphocytes secrete antibodies upon antigen recognition/activation, which then perform a number of functions, including opsonization, neutralization, agglutination, and complement activation [76].

### 3.2. B Lymphocytes

B lymphocytes are the producers of B-cell receptors and antibodies, the membrane and secreted forms of immunoglobulins (Igs) [76,77]. The majority of work to date studying the effects of suboptimal temperatures on these cell populations has been performed using channel catfish as a model. The magnitude of PBL proliferation in channel catfish in response to the B cell mitogen LPS was found to be relatively independent of the temperatures studied *in vitro*, but peak proliferation was delayed as the assay temperature decreased, with the greatest delay at 17°C relative to 22°C, 27°C, and 32°C [78]. However, when PBLs were isolated from fish maintained at 11°C compared to 24°C, proliferation was inhibited at both assay temperatures, 17°C and 27°C [79]. Additionally, the number of B cells in the blood decreased following exposure to 11°C, and did not recover until 5 weeks later, implying that channel catfish may decrease circulating B cells in response to large temperature decreases, but that they may be able to acclimate to the lower water temperature over time. Increases in unsaturated fatty acids in the plasma membrane have also been observed in B cells from fish acclimated to 17°C versus 24°C *in vivo*, and while the effect this change has on lymphocyte function has not been elucidated, the authors speculated that it stiffened cell membranes, decreasing cell-to-cell interactions and thus immune responses [80].

Studies in rainbow trout have also provided evidence of suboptimal temperatures adversely impacting B lymphocyte function. An increase in transcript expression was observed at 15°C for all B cell markers studied (secretory *igm*, membrane-bound *igt*, *pax5*, and *blimp1*) in the anterior kidney post-infection with *Tetracapsuloides bryosalmonae*, and for all but IgT in the posterior kidney, with *blimp1*, the B cell differentiation marker being most strongly up-regulated [34]. However, no significant up-regulation was observed at 12°C, indicating that B cell activation in response to pathogens may be impaired at low temperatures. Additionally, a significant increase in posterior kidney IgM^+^ B cells was observed at seven weeks post-infection at 15°C relative to 12°C. Similar findings in another study showed that in response to infection with *Aeromonas salmonicida*, the increase in the percentage of B cells in the spleen and the blood was larger and happened more rapidly at 16°C compared to 11°C, which suggests that B lymphocyte proliferation may also be impaired under these conditions [31].

Of note is the potential impact of rapid, large temperature decreases on B lymphocytes and the teleost immune system as a whole. A drop in temperature from 25°C to 16°C over 3 h resulted in a significant reduction in the percentage of B cells in the spleen and blood of the common carp, that recovered after 24 h [32]. An increase in annexin V-positive B cells in the blood was observed immediately following the temperature shock, implying that there may be a reduction in circulating B cells in response to acute stress. While water temperature fluctuations in the wild do not occur as quickly as the changes used in these experiments, it is important to be aware of the potential for large temperature decreases to act as short-term stressors and immunosuppressors in both *in vivo* and *in vitro* studies.

### 3.3. T Lymphocytes

Suboptimal temperature effects on T cells have been most thoroughly studied in the channel catfish. Similar to B cell studies, T cell proliferation in response to the T cell mitogen concanavalin A was inhibited, and the number of T cells in the blood were decreased when PBLs were isolated from fish maintained at 11°C compared to 24°C [79]. However, unlike in B cells, peak T-cell proliferation in PBLs stimulated with concanavalin A was proportional to temperature, and was greatly reduced at 22°C relative to 27°C and 32°C [78]. Furthermore, mixed leukocyte reactions using PBLs from 24°C-acclimated fish were also temperature-dependent with respect to time, with the fastest reaction occurring at 27°C, and the slowest at 17°C [81]. In a later study, monocyte cell lines pulsed with antigen at 11°C, 17°C, or 27°C were able to induce proliferation of autologous responder T cells from PBLs, although peak proliferation was again delayed at the lower temperatures [82]. An increase in antigen-presenting cell-associated radioactivity due to uptake of radiolabelled antigen was observed at all temperatures, suggesting that the observed suppression of T cell responses at the suboptimal temperatures was not due to impaired antigen presentation by the antigen-presenting cells. Further studies attempted to explain the differential effects of low temperature on the magnitude of channel catfish T and B cell proliferation through the study of fatty acids in these cells. As seen in B cells, unsaturated fatty acid levels in the plasma membrane of T cells were increased at 17°C versus 24°C [80]. However, B cells appear to be able to synthesize oleic acid endogenously from stearic acid while T cells cannot, and accumulate stearic acid in their membranes which decreases membrane fluidity. This difference may explain the different effects of low temperatures on lymphocyte proliferation [83]. Following addition of exogenous oleic acid to T cells, 60% of the proliferation response to concanavalin A was recovered at the lower temperature, further supporting this idea [83]. 

Inhibitory effects of suboptimal temperatures have also been observed in other fishes, often resulting in decreases in T cell activation and activity. For example, T lymphocyte proliferation in PBLs appears to be proportional to temperature in the common carp, with proliferation increasing from 12°C to 20°C to 28°C both *in vivo* and *in vitro* [38]. Additionally, in the ginbuna crucian carp, cell-mediated cytotoxicity, believed to be due to specific cytotoxic T cells, was more efficient when cells were cultured at 25°C versus 20°C or 15°C, suggesting that this process may also be temperature-dependent [84]. However, while specific cell-mediated cytotoxicity was downregulated at 9°C versus 18°C in the common carp, a decrease in activity was also observed at 26°C, which may act as a nonpermissive higher temperature for carp [85]. Macrophage activating factor, now known as interferon gamma (IFN-γ), production from T cells in rainbow trout head-kidney leukocytes was lower in cells held at 6°C compared to 10°C and 18°C, although supernatants from each temperature condition were still able to significantly affect the respiratory burst activity of macrophages [42]. Interestingly, when leucocytes were collected from fish acclimated to 6°C and then allowed to acclimate for 48 h to the higher assay temperatures, some of the IFN-γ activity could be recovered, indicating that detrimental effects of suboptimal temperatures on T cells may be reversible. Overall, these data seem to suggest that suboptimal temperatures negatively impact T cell proliferation and activity in fish, which may adversely affect their ability to limit the spread of infection and mediate specific responses against pathogens.

Polarization of helper T (T_H_) cells results in the induction of T_H_1 and T_H_2 cells, which mediate type I and type II immunity respectively [86]. T_H_1 cells mediate macrophage activation and enhanced cytotoxic T cell activity, important for responding to intracellular bacteria, while T_H_2 cells are important effector cells against extracellular parasites due to their ability to activate mast cells and eosinophils. There is some experimental evidence that suboptimal temperatures may affect the polarization process in rainbow trout. Following exposure to *Tetracapsuloides bryosalmonae*, T_H_1 associated genes were mildly upregulated at the cold-stress temperature of 12°C in rainbow trout kidney leukocytes, while T_H_2 associated genes were not [34]. However, both sets of genes remained upregulated for 2 weeks post-exposure at the control temperature of 15°C. By 6 weeks post-exposure, only the T_H_2 associated gene expression was still increased, indicating that suboptimal temperatures may be able to impact immune response polarization.

### 3.4. Antibodies and the Humoral Response

Suboptimal temperature impacts on antibody production have been reported in numerous fish species, with B cell responses to T-dependent antigens affected more appreciably. In channel catfish, the magnitude of the peak response of IgM secreting cells isolated from PBLs was only proportional to the assay temperature for dinitrophenol (DNP) conjugated to keyhole limpet hemocyanin (KLH), a T-dependent antigen, and was temperature-independent for the T-independent antigen, trinitrophenol-LPS [82,87]. Additionally, the peak response was delayed for both antigens as the temperature decreased, with the largest delay occurring at 17°C versus 22°C, 27°C, or 32°C. A similar observation was seen in carp, as antibody production in response to DNP-KLH was proportional to temperature, with antibody levels at 12°C significantly lower than at 20°C or 28°C [37]. However, this pattern of temperature effects on antibody production is not always observed. For example, following an *in vivo* drop in temperature from 25°C to 16°C over 3 h, decreases in antibody production in response to both DNP-KLH, and trinitrophenol-LPS were observed [32]. Additionally, mucosal immunoglobulin production in channel catfish after bath vaccination with DNP-KLH was suppressed at the warmer temperature of 30°C compared to 23°C and 15°C, in contrast to the serum immunoglobulin response [88]. These studies suggest that the effect of low temperatures on the ability of fish to produce antibodies against T-dependent antigens may be greater than it is for T-independent antigens, except perhaps in the cases of rapid, large temperature reductions and mucosal responses.

Natural antibodies are important mediators of nonspecific immunity that help provide protection against pathogens, and are found at high levels in fish serum [19]. Basal serum levels of these antibodies appear to be differentially affected in different fish species by low temperatures. In rainbow trout, serum immunoglobulin levels in the summer were higher than in the winter in fish larger than 1 kg from the same fish farm, with mean seasonal temperatures of 19°C and 7°C respectively [6]. Conversely, no differences in basal plasma IgM levels were observed in tilapia maintained at 25°C or 12°C over 15 days, although perhaps differences in basal plasma IgM levels would have been observed over a longer time interval [89]. More data is needed to determine the effect of environmental temperature decreases on basal immunoglobulin levels, although current results indicate that the outcome is species-dependent.

Suboptimal temperature impacts on antibody production in response to live or inactivated pathogens and vaccines is also varied, although generally suppressive. Following infection with *Ichthyophthirius multifiliis*, serum antibody levels in channel catfish were significantly higher at 25°C and 30°C relative to 15°C and 20°C, with no detectable specific antibodies after 21 days at 15°C [90]. Additionally, serum antibody titres following injection of inactivated *Yersinia ruckeri* or phosphate-buffered saline were also temperature-dependent, with the lowest titres observed at 5°C versus 15°C and 25°C, indicating that humoral responses against inactivated pathogens, as well as in response to injection, are suppressed by cold temperatures [28]. A similar result was observed in rainbow trout when a DNA vaccine against VHSV was employed. A higher serum antibody titre was observed at 15°C than at 10°C, and no detectable antibodies were present at 5°C [91]. Furthermore, the population percentage survival upon subsequent exposure to the virus was also temperature-dependent, suggesting that vaccination efficiency may be decreased at lower temperatures. However, production of specific antibodies to inactivated *Aeromonas salmonicida* appeared to be delayed at the normal temperature of 15°C compared to 12°C in rainbow trout, with both reaching similar levels by 90 days post stimulation, showing that the effects of suboptimal temperatures are not universally similar [31]. Overall, these data indicate that the effects of suboptimal temperatures on antibody production in response to live or inactivated pathogens and vaccines is generally suppressive, potentially compromising the ability of the fish to clear infections effectively at these temperatures.

## 4. Concluding Remarks and Future Challenges

The effect of environmental temperature on teleost immune systems thus varies depending upon the duration and magnitude of the temperature change and the fish species examined, since teleosts have adapted to a wide variety of environmental temperatures. In general, lower temperatures lead to a shutdown or slowing of immune response mechanisms, which is generally reversible upon return to warmer temperatures, suggestive of overwintering strategies. Components of the innate immune system had varied responses to cold stress, with the enhancing of innate cellular components potentially providing partial compensation for deficiencies in adaptive immunity, as there was a somewhat consistent suppression of the adaptive immune system in response to colder temperatures. This suppression could be linked to some of the detrimental impacts of low temperatures on other aspects of the innate immune system, as they are required to induce adaptive immunity. These effects could lead to a decreased ability of fishes to respond to pathogens over the winter months or in response to temperature decreases, negatively impacting their health. However, this tradeoff may be energetically favorable to the fish host at low temperatures, comparable to the diminished immune responses seen in hibernating mammals [92], and this may be why the effects of cold temperatures on the immune system appear to be generally conserved.

There are a number of challenges in attempting to study the effects of suboptimal temperatures on the teleost immune system. The use of different model systems with different thermal optimums and preferences makes it challenging to compare the magnitude of temperature decreases in different experiments, as a change as small as 3°C may negatively impact one fish species, and yet have no effect on many others. Additionally, the acclimation conditions can vary quite significantly, ranging from rapid large temperature decreases to rearing fish at a specific temperature for a period of months. Hence, it is not surprising that while rapid decreases in temperature appear to be overwhelmingly detrimental, fish subjected to long-term acclimation generally appear to be somewhat better adapted to deal with cold temperatures. Another important consideration is the assay temperature employed, as a number of studies focusing on different immune parameters have reported that peak response activity is observed when assay temperature equals fish acclimation temperature. However, this aspect is often unreported in the literature, making it challenging to fully comprehend the results of such studies. While some of these discrepancies are tougher to address practically than others, it is important that future studies attempt to take them into account to try to make it easier to compare different experimental studies.

A final consideration for future studies is the value of taking more holistic experimental approaches. While basal levels of immune components are important for dealing with initial pathogen interaction, inducible elements are equally important and necessary for clearance of infectious agents, yet these two facets are not often examined in the same study. Additionally, the virulence of pathogens is also impacted by temperature [18], although it can be difficult to tease apart these effects from those on the host immune system. Notwithstanding, it is important to acknowledge the potential value in performing more intensive and comprehensive studies that can strengthen our understanding of the impact of low environmental temperatures on host-pathogen systems, specifically in elucidating the underlying immune mechanisms that result in host mortality when fishes are exposed to acute or chronic cold stress.
